# [Corrigendum] Sulforaphane sensitizes human cholangiocarcinoma to cisplatin via the downregulation of anti‑apoptotic proteins

**DOI:** 10.3892/or.2023.8646

**Published:** 2023-10-11

**Authors:** Rokas Račkauskas, Dachen Zhou, Simonas Ūselis, Kęstutis Strupas, Ingrid Herr, Peter Schemmer

Oncol Rep 37: 3660–3666, 2017; DOI: 10.3892/or.2017.5622

Following the publication of this article, an interested reader drew to the authors' attention that, in Figs. 3 and 4A on p. 3664, the respective β-actin controls for the cell lines TFK-1 and HuCCT-1 appeared to have mixed up, comparing the western blots between the two figures.

After re-examining their data, the authors have realized that the control blots in Fig. 4A were inadvertently presented the wrong way around. The corrected version of Fig. 4, showing the correctly presented western blotting data in Fig. 4A, is shown on the next page. Note that this error did not grossly affect the results or the conclusions reported in this paper. The authors sincerely apologize for the error that was introduced during the preparation of this figure, and thank the Editor of *Oncology Reports* for allowing them the opportunity to publish a corrigendum. Furthermore, they regret any inconvenience caused to the readership.

## Figures and Tables

**Figure 4.**
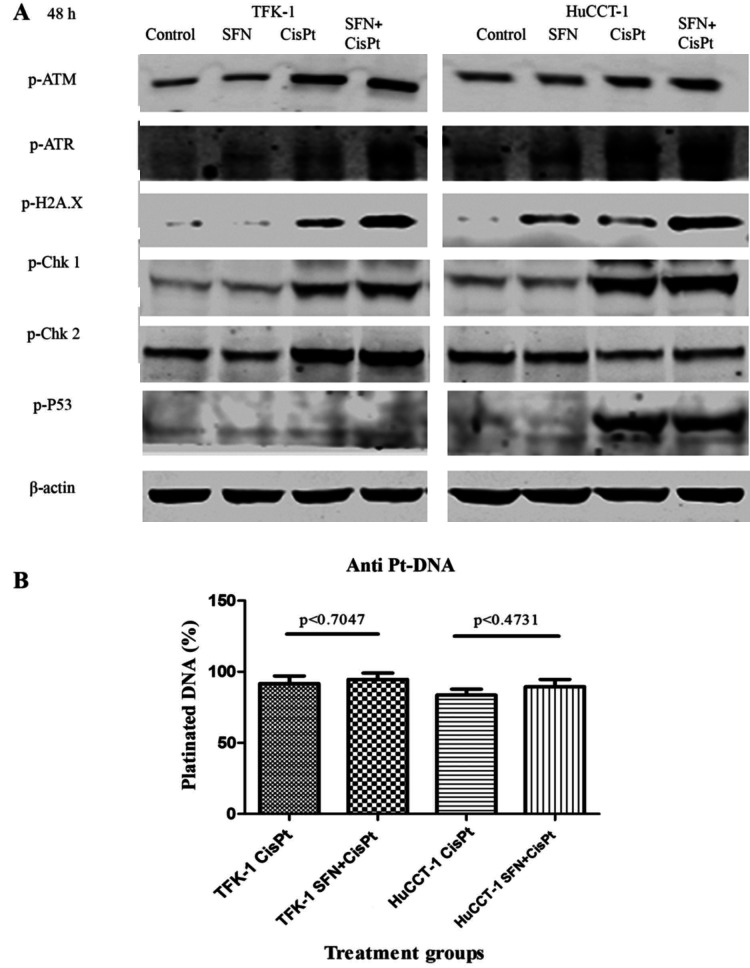
Impact of sulforaphane on DNA damage and checkpoint activation. (A) Cell lines were treated with sulforaphane (SFN), cisplatin (CisPt) and SFN+CisPt as described in Materials and methods. The expression levels of phospho(p)-ATM, p-ATR, p-H2A.X, p-P53, p-Chk1 and p-Chk2 were measured using western blotting. (B) Flow cytometry was used to quantify platinated DNA after treatment with CisPt and SFN+CisPt. Data are shown as mean ± SD.

